# Vaccination trends and operational challenges in Peste des Petits Ruminants eradication in Ethiopia

**DOI:** 10.1038/s41598-026-41404-7

**Published:** 2026-02-27

**Authors:** Enyiew Alemnew Alamerew, Thomas Cherenet, Fasil Aklilu, Zelalem Yitayew, Derib Aydefruhim, Firdawok Ayele, Anmaw Shite Abat

**Affiliations:** 1 Amhara Agricultural Research Institute, Debre Birhan Agricultural Research Centre, Debre Birhan, P.O. Box 112, Ethiopia; 2 Department of Veterinary Pathobiology, University of Gondar, Gondar, Ethiopia; 3 Livestock and Fishery Sector Development Project, Ministry of Agriculture, Addis Ababa, Ethiopia; 4 Department of Serology, Animal Health Institute, Sebeta, Ethiopia; 5 Livestock and Fishery Office, Diseases Control and Prevention, Debre Birhan, Ethiopia

**Keywords:** Constraints, North Shewa Zone, Progress towards eradication, Risk-based vaccination strategy, Small ruminants, Diseases, Health care, Immunology

## Abstract

Peste des Petits Ruminants (PPR) is a major transboundary disease in Ethiopia, where the national goal is eradication by 2027. However, evidence on vaccination performance and operational challenges remains limited. This study assessed vaccination trends over six years (September 2018–August 2024) and conducted surveys with animal health professionals (AHPs) (January–May 2024) to identify key enabling factors and constraints influencing eradication efforts in the North Shewa Zone. The findings revealed that a risk-based vaccination strategy (RBVS) has been implemented since 2018, focusing on high-risk areas and employing rapid antigen detection tests to confirm outbreaks. Once an outbreak is confirmed, AHPs collaborate with regional coordinators to isolate at-risk populations and administer a live attenuated PPR vaccine. Over the study period, 62 vaccination campaigns were conducted, averaging 10.3 campaigns per year. The highest number occurred in 2019 (16 campaigns, 25.81%), while the lowest was in 2022 (one campaign). Kewet district recorded the highest number of campaigns (six), whereas six districts received no vaccinations. A total of 2,917,330 small ruminants were vaccinated, with Minjar-Shenkora district reporting the highest number (384,989 animals). The year 2023 saw the peak in vaccination coverage, with 1,284,339 animals vaccinated. The study identified factors affecting eradication efforts, with key enablers including a national policy (82.6%), sufficient vaccine production (71.74%), and adequate vaccination tools (80.43%). Major challenges included budget constraints (84.78%) and security concerns (65.22%), along with operational issues such as lack of animal identification, absence of post-vaccination sero-monitoring (PVS), high herd mobility, and rapid turnover of young stock. In conclusion, these challenges may compromise vaccination effectiveness and hinder eradication efforts. Nevertheless, strong regional commitment and enabling factors provide a promising foundation for progress. To enhance PPR control and achieve eradication, the study recommends strengthening surveillance, improving post-vaccination sero-monitoring, optimizing vaccine distribution, and addressing key operational barriers.

**Supplementary Information:**

The online version contains supplementary material available at 10.1038/s41598-026-41404-7.

## Introduction

PPR is a highly contagious transboundary viral disease most commonly reported in domestic sheep and goats and remains one of the most significant constraints to small ruminant production in endemic regions worldwide^1,2^. The disease is caused by PPR virus (PPRV), a member of the genus *Morbillivirus*, and is clinically characterized by fever, oral erosions, diarrhea, respiratory distress, and pneumonia, often resulting in high morbidity and mortality. In susceptible domestic populations, PPR can affect up to 100% of animals, with mortality rates ranging from 10% to 90%^3–5^. Beyond its impact on livestock, PPR also poses a serious threat to wildlife conservation and biodiversity, with outbreaks in susceptible wild ungulates causing population losses of up to 80%^6^. Although impacts on African wildlife are less clearly documented, several vulnerable species remain at risk^7^; in Ethiopia, for example, critically endangered wildlife such as the Simien ibex could face substantial conservation threats following spillover from domestic animals.

Since its first recognition in West Africa in 1942, PPR has spread extensively across Africa, the Middle East, and Asia and is now endemic in more than 70 countries that collectively host the majority of the global small ruminant population^3,8–12^. In Ethiopia, where PPR is endemic, reported prevalence over the past decade has shown marked spatial variability, ranging from 2.1% to 75.5% across different regions and production systems^13,14^. Notable prevalence estimates include 46.53% in the Tigray Region^15^, 75.5% in Asossa Zone^14^, 12.9% in Bale Zone^16^, 60.15% in Afar Region^17^, 32.5% in Northwest Ethiopia^18^, 10.3% in Dera and Gerar Jarso Districts of Oromia^19^, 32.1% in Borena Zone^20^, and 60.8% in the Amhara Region^21^.

The socioeconomic impact of PPR is particularly severe in Ethiopia, where sheep and goats play a central role in household income generation, food security, and livelihood resilience^4,22^. The disease causes significant losses through morbidity and mortality^23,24^, reduced productivity, market disruptions, and movement restrictions, with global annual economic losses estimated at approximately USD 2.1 billion^25^. Field studies in Ethiopia have reported flock-level morbidity rates of 83% in sheep and 87% in goats, with mean economic losses per affected flock estimated at ETB 7,835 (USD 329) for sheep and ETB 7,136 (USD 300) for goats. During outbreaks, average losses amounted to ETB 319 (USD 13.4) per sheep and ETB 306 (USD 12.9) per goat, with mortality accounting for more than 70% of total losses. In contrast, vaccination costs remain relatively low, estimated at approximately ETB 3 per correctly vaccinated animal (20).

Given its transboundary nature, extensive geographic distribution, severe economic consequences, and the availability of a safe and effective vaccine, PPR has been prioritized for global eradication^26,27^. Accordingly, the Food and Agriculture Organization of the United Nations (FAO) and the World Organization for Animal Health (WOAH) launched a global strategy for the control and eradication of PPR, including the PPR Global Eradication Programme (GEP), with the goal of achieving worldwide eradication by 2030^8,28^. The strategy emphasizes sustained, risk-based vaccination using a live-attenuated vaccine that provides long-lasting immunity, supported by strengthened surveillance systems and targeted interventions in high-risk populations and areas^9,12,29^.

Ethiopia has aligned its national PPR control and eradication strategy with this global framework and has set an ambitious target to eliminate PPR by 2027^30^. Vaccination remains the cornerstone of this strategy, with increasing implementation of RBVS focusing on outbreak-prone districts and areas characterized by high animal movement. Despite these efforts, PPR continues to be endemic, with recurrent outbreaks reported across multiple regions and production systems^4,31^. Between 2018 and 2022 alone, more than 550 outbreaks were reported nationally, underscoring persistent challenges related to vaccination coverage, campaign implementation, and post-vaccination monitoring^20^.

Within this national context, the North Shewa Zone of the Amhara Region represents a critical epidemiological and operational setting for evaluating progress toward PPR eradication. The zone hosts a large and mobile small ruminant population estimated at 2,738,402 animals, contributing substantially to Ethiopia’s national small ruminant population of 90,396,066^32^. The presence of diverse production systems and frequent animal movements further facilitates PPRV transmission^24,33^. Although RBVS have been implemented in the zone since 2018, repeated outbreaks, 48 confirmed between September 2018 and August 2024, continue to occur^24^, indicating potential shortcomings in campaign frequency, spatial coverage, and operational execution. Effective eradication requires not only vaccine availability but also consistent and equitable campaign delivery, adequate financial and logistical resources, secure operational environments, and strong institutional coordination^8,9^. Despite the importance of these factors, to the authors’ knowledge, evidence from Ethiopia, particularly from North Shewa, on long-term vaccination performance, the spatial distribution of vaccination campaigns, and the operational enablers and constraints within the FAO–WOAH GEP Stepwise PPR Monitoring Framework (SPMF) remains limited. This knowledge gap constrains data-driven evaluation of ongoing eradication efforts and hampers informed programmatic adjustments^24,32–35^. In response to these persistent challenges and the lack of comprehensive evidence on vaccination trends, the present study was undertaken to assess the trend of the RBVS and to identify key enabling factors and constraints influencing PPR eradication efforts in the study area.

## Materials and methods

### Study area

**Fig. 1:**
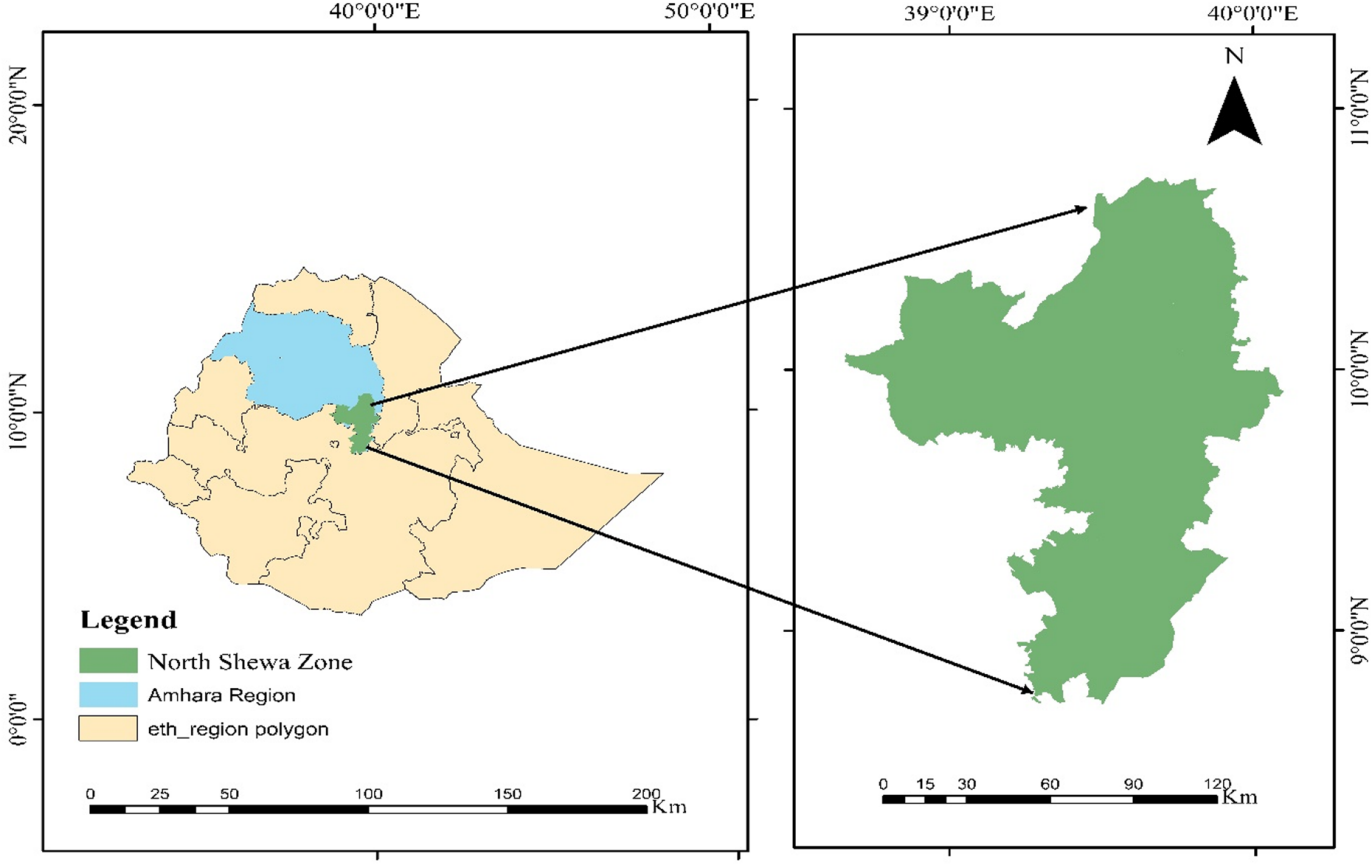
Map of the study areas.

The study was carried out in all 24 districts of the North Shewa Zone, Amhara National Regional State, Ethiopia (Fig. 1). Geographically, the zone lies between 9° and 11° N latitude and 38° and 40° E longitude, covering an area of approximately 15,936 km²^36^. The zone exhibits considerable altitudinal variation, ranging from about 1,500 m above sea level in the lowland areas to more than 3,000 m in the highlands. This elevation gradient gives rise to diverse agro-climatic conditions. Temperatures in the highland areas typically range from 10 °C to 20 °C, while lowland areas experience higher temperatures of approximately 20–30 °C. The zone experiences a main rainy season from June to September, a shorter rainy period from February to May, and a dry season from October to January. Mean annual rainfall across the zone ranges between 1085 mm and 1199 mm^37^.

### Study population

The study population consisted of sheep and goats originating from all districts of the North Shewa Zone. The zone is characterized by a large small ruminant population, estimated at 2,738,402 sheep and goats, ranking second within the region and contributing substantially to Ethiopia’s total small ruminant population of 90,396,066^32^. The animals were owned by smallholder farmers as well as governmental and non-governmental organizations. Smallholder-owned flocks were managed predominantly under extensive production systems, involving daytime grazing on communal lands, fallow areas, and crop residues, with minimal or no feed supplementation and overnight housing. In contrast, sheep and goats managed by governmental and non-governmental organizations were raised under semi-intensive systems that combined grazing with routine supplementary feeding.

The target population also comprised AHPs directly involved in PPR prevention and control, including zonal and district veterinary officers and frontline animal health workers responsible for planning, supervising, and implementing vaccination activities. Only personnel who had participated in at least one PPR vaccination campaign since September 2018 were included to ensure practical field-based experience. Where veterinarians covered more than one district, vaccination data were reported in aggregated form based on official responsibilities and verified records. Questionnaire items focused on respondents’ professional roles in vaccinating sheep and goats, with wording revised to avoid ambiguity regarding personal vaccination status.

### Study design and sample size determination

A retrospective analysis was conducted to evaluate PPR vaccination trends using secondary data collected from September 2018 to August 2024. This was complemented by a cross-sectional study aimed at examining key enabling factors and constraints influencing PPR eradication, using questionnaires administered to AHPs between January and May 2024. Participants were recruited from five districts, Debre Berhan, Basona-werena, Kewet, Shewa-robit, and Menz-mama, selected based on security conditions, road accessibility, prior experience with at least one PPR vaccination campaign. Eligible participants were veterinarians with direct involvement in PPR vaccination campaigns. A purposive sampling approach was employed to ensure inclusion of veterinarians with relevant field experience. Potential participants were identified through district veterinary offices and invited to participate after being informed about the study objectives and procedures, and those who provided informed consent were enrolled. The sample size for the expert survey was determined using a finite population formula commonly applied in expert-based studies^38^:

$$\:n=\frac{N}{1+N\left({e}^{2}\right)}$$

where N = the population size (*N* = 86), e = margin of error (e = 10), Accordingly, a total of 46 experts were included in the study.

### Data collections

#### Questionnaire survey

Data for this study were collected using structured, semi-structured, and open-ended questionnaires designed to identify the major constraints affecting the progress of the PPR eradication program through RBVC (SI Annex 2). A total of 46 AHPs from the study areas participated in the data collection. The primary objective was to identify the enabling factors and determinants influencing the success of the RBVC and to propose corrective measures based on the findings. Before conducting the interview’s, informed consent was obtained from each respondent, and the objectives of the study were clearly explained. Data were collected through face-to-face interviews, and the questionnaire was pre-tested in a nearby area to ensure clarity and appropriateness.

In addition to the questionnaire survey, key informant interviews were conducted with zonal PPR eradication coordinators and senior animal health experts to document implementation modalities of the RBVS, including training procedures, outbreak confirmation, and vaccination approaches under different security contexts. Information from these interviews was summarized thematically and used to describe programmatic implementation (SI Annex 1b).

#### Retrospective data: PPR vaccination trends

Data for this study were collected using structured and semi-structured questionnaires. It focused on evaluating the RBVS, gathering information on the start years of PPR-RBVS, vaccination approach, and the number of small ruminants vaccinated annually. This data covered the period from the start of the RBVS to the data collection period across all districts in the North Shewa Zone. A six-year retrospective dataset on PPR vaccination was obtained from the North Shewa Zone Animal Health Office through the Disease Outbreaks and Vaccination Activity Reports (DOVAR) system. The data, organized by year and district, include details on vaccination figures (number of sheep and goats vaccinated) (Supplementary Information (SI) Annex 1a).

### Data management and statistical analysis

Data from retrospective records and questionnaire surveys were entered into Microsoft Excel and analyzed using STATA version 17. The datasets were cleaned and validated for completeness, consistency, and accuracy prior to analysis, with discrepancies resolved by cross-checking original records and field notes. Descriptive statistics, including frequencies and percentages, were used to summarize PPR vaccination coverage by year and district. Time-series graphs were generated to illustrate vaccination trends over the six-year study period. The spatial distribution of PPR vaccination campaigns was analyzed using ArcGIS software version 10.5 to map vaccination intensity and geographic coverage across the North Shewa Zone.

Questionnaire data were analyzed descriptively to assess constraints and enabling factors affecting the effectiveness of the RBVS, with frequencies and percentages summarizing responses across districts. Open-ended responses from animal health professionals were analyzed using thematic content analysis. Expert statements were systematically coded and grouped into categories and overarching themes, and the occurrence of each theme was summarized to aid interpretation. Representative quotations were included to illustrate key themes and preserve the original meaning of expert opinions. Data obtained from key informant interviews were analyzed qualitatively using thematic content analysis. Interview notes were reviewed and organized by topic areas defined in the interview guide, including training practices, outbreak confirmation procedures, vaccination implementation, and security-related adaptations. Responses were coded manually, and recurrent themes were identified through iterative reading. Key themes were summarized to describe the implementation of the RBVS in the study area.

## Results

### Implementation of risk-based vaccination within the stepwise framework

Information on the implementation of the RBVS was obtained through key informant interviews. According to respondents, RBVS has been implemented in the zone since September 2018. Pre-campaign training and awareness sessions are routinely provided to district- and PA-level animal health professionals and local coordinators, typically one day before vaccination activities. These sessions cover disease distribution, vaccine handling and transportation, administration procedures, and data recording. Informants further reported that periodic training is conducted at zonal, district, and PA levels, focusing on PPR case definitions, participatory disease surveillance, outbreak reporting, morbidity and mortality assessment, field diagnostic techniques (rapid antigen detection), and vaccination coverage. Experts indicated that suspected outbreaks are confirmed using rapid antigen detection tests in collaboration with regional coordinators, after which at-risk populations are isolated and vaccinated using live attenuated PPR vaccine. Vaccination delivery approaches vary according to security context: in stable areas, joint teams from multiple PAs conduct campaigns, whereas in insecure areas, vaccination is performed individually by local animal health professionals within their respective PAs. This flexible strategy ensures continued progress toward eradication despite local challenges.

**Fig. 2:**
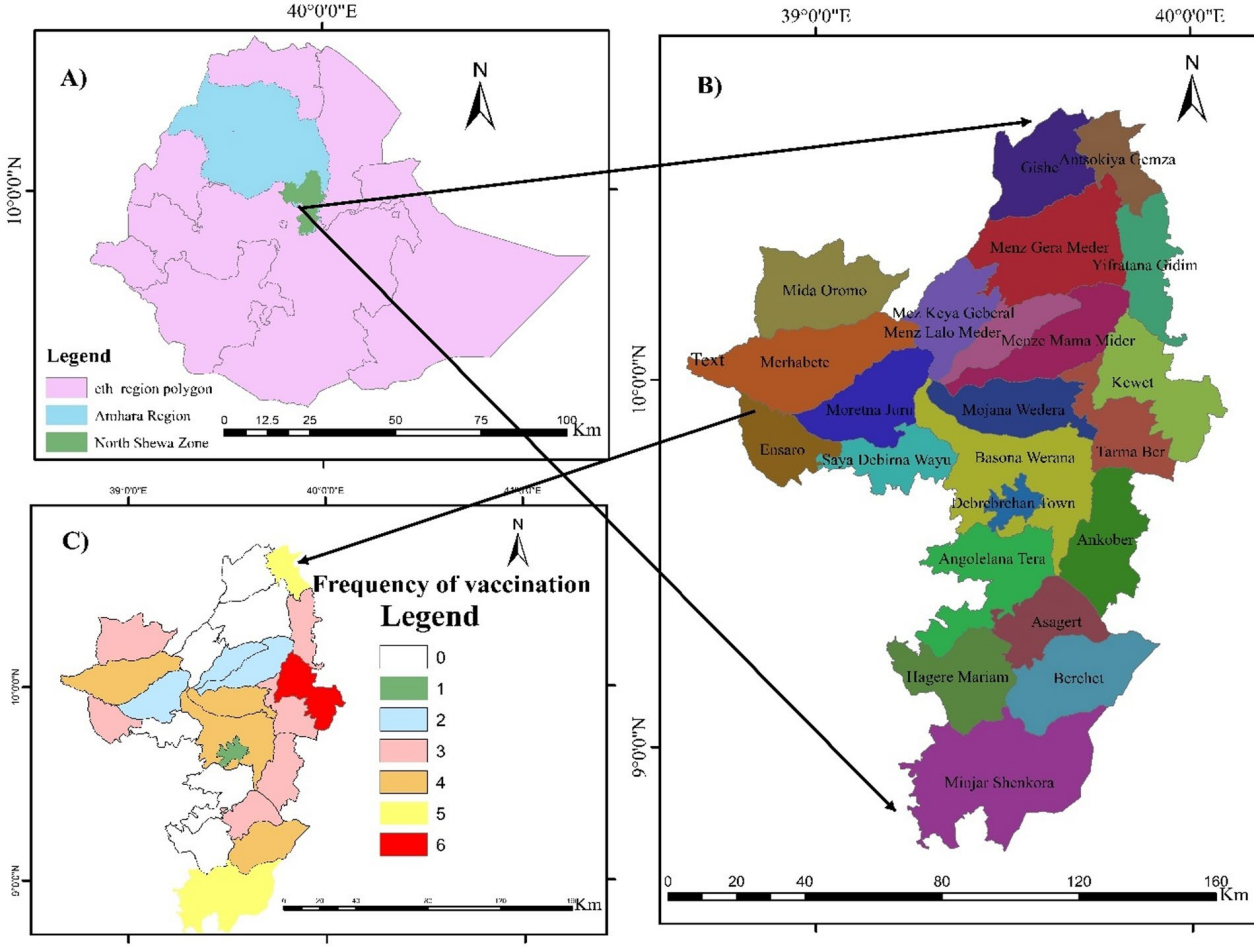
Frequency of PPR vaccination campaigns in the study districts from 2018 to 2024. Legend numbers (0–6) indicate the total number of vaccinator visits per district across the study years.

During the study periods, a total of sixty-two vaccinations campaigns were recorded in the study zone. Kewet District had the highest vaccination campaigns (6) accounting for 10% of all reported vaccinations campaigns. Close behind were Antsokiya-gemza, Minjar-shenkora, and Shewa Robit districts, each with five vaccinations campaigns, collectively representing 25% of the total. In contrast, six districts; Menz-gishe, Menz-keya, Menz-gera, Angolelana-tera, Hagere-mariam, and Siya-wayu, did not receive any vaccinations during this period (Fig. 2).

Overall, 2,917,330 small ruminants were vaccinated, including 1,284,339 sheep and 1,632,991 goats. The Minjar-shenkora district had the highest number of vaccinated animals at 384,989, followed by Kewet with 363,302 (Table 1), further demonstrating the zone’s commitment to controlling and eradicating the disease.

**Table 1:** Number of small ruminants vaccinated through PPR-RBVC in study districts.

Study districts	Number of small ruminants vaccinated		
	Sheep	Goats	Total
Debre-birhan	3,948	52	4000
Basona-werana	172,514	57,771	230,285
Ankober	19,075	36,525	55,600
Mojana-wedera	105,890	35,994	141,884
Taramaber	136,327	180,822	317,149
Kewet	64,152	299,150	363,302
Shewa-robit	7,949	43,398	51,347
Efratana-gidim	48,558	58,456	107,014
Antsokiya-gemza	112,619	113,934	226,553
Menz-lalo	53,300	20,850	74,150
Menz-mama	156,512	52,740	209,252
Asagirt	35,700	36,800	72,500
Minjar-shenkora	180,503	204,486	384,989
Berehet	34,928	101,110	136,038
Moretna-jiru	1,250	3,200	4450
Ensaro	19,810	36,490	56,300
Merhabete	44,055	188,533	232,588
Mida-weremo	87,249	162,680	249,929
Total	1,284,339	1,632,991	2,917,330

**Fig. 3:**
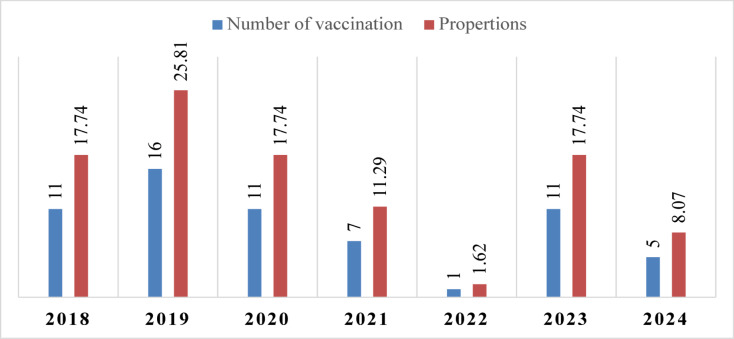
Frequency of PPR vaccination campaigns during the study period. The vaccination number (1–16) represents the total number of vaccinator visits per year across all districts.

Although the study period included data from 2018 to 2024, data for these two years were incomplete. Nonetheless, the results show an average frequency of 10.3 vaccination campaigns per year. The highest frequency was observed in 2019, with 16 campaigns, accounting for 25.81% of the total during the study period. This was followed by 2018, 2020, and 2023, each with 11 campaigns, collectively representing 52.2% of all campaigns. In contrast, 2022 had the lowest frequency, with only 1 campaign, representing 1.61% of the total (Fig. 3).

During the study period, the highest number of small ruminants vaccinated was in 2023, with a total of 1,284,339, comprising 483,052 sheep and 535,088 goats. In contrast, 2022 recorded the fewest vaccinations during the full study period from 2019 to 2023, with only 49,550 small ruminants vaccinated, comprising 17,010 sheep and 32,540 goats. Notably, in 2024, a total of 132,691 small ruminants were vaccinated, despite it not being a complete study year (Table 2).

**Table 2:** Number of small ruminants vaccinated during the study periods in North Shewa Zone.

Study years	Number of small ruminants vaccinated		
	Sheep	Goat	Total
2018	10,200	0	10,200
2019	269,275	313,415	582,690
2020	250,612	315,519	566,131
2021	194,910	363,018	557,928
2022	17,010	32,540	49,550
2023	483,052	535,088	1,018,140
2024	59,280	73,411	132,691
Total	1,284,339	1,632,991	2,917,330

### Enabling factors and constraints to stepwise progress

This study assessed factors influencing RBVS effectiveness, with 82.6% of respondents acknowledged the presence of a strong national PPR eradication policy. Vaccine production capacity was also viewed favorably, with 71.74% reporting that national laboratories are capable of producing sufficient quantities to meet demand. Additionally, 80.43% indicated that vaccination tools, such as syringes and needles, were adequately supplied. The presence of farmers’ organizations, reported by 65.22%, is seen as a vital asset for mobilizing community support. Moreover, 65.22% noted that PPR eradication efforts are integrated with the control of other small ruminant diseases, such as internal and external parasite management, reflecting a holistic approach to animal health. In support of technical capacity, 60.87% of respondents reported adequate data analysis ability, reflecting their actual use of collected data for statistical analysis and application of results in evidence-based vaccination service delivery (Table 3).

Despite these strengths, major challenges were identified, with 84.78% of respondents reporting budget constraints affecting vaccination campaigns, particularly for logistics such as per diem, transport, data recording materials, and health insurance, under challenging field conditions. Security concerns were reported by 65.22%, hindering field operations and limiting vaccination coverage. Trust and transparency issues with national authorities were noted by 47.83% of respondents, assessed through their confidence in vaccine efficacy, service delivery, and the accuracy and timeliness of information provided. Organizational effectiveness was another area of concern, with 65.22% indicating that eradication measures were inadequately coordinated and lacked strategic coherence (Table 3).

Infrastructure limitations persisted, with 47.83% of respondents rated storage capacity as limited, which could compromise vaccine effectiveness. There were also serious shortages in critical logistics, including vehicles and cold chain equipment. Regarding veterinary infrastructure, findings were mixed. While 60.87% reported adequate capacity for data interpretation, major gaps were noted in sample collection and transportation. Human resources were also a limiting factor, only 41.3% of respondents believed there were sufficient personnel in both number and skill, while nearly half rated the available human resources as only moderate (Table 3).

**Table 3:** Descriptive statistics of major constraints of PPR eradication program.

S. No.	Question	Responses	Frequency (%)
1)	Is there a national policy for PPR eradication?	Yes	82.6
		No	17.4
2)	Do national authorities provide transparent information regarding the PPR situation?	Yes	47.83
		No	52.27
3)	Do you think that PPR eradication measures are organized and implemented in a coherent way?	Yes	34.78
		No	65.22
4)	Do you have sufficient national budget allocation for PPR eradication?	Enough	10.87
		Limited	84.78
		Non-existent	4.35
5)	Are there national laboratories capable of producing sufficient vaccines to meet the vaccination targets of the National Strategic Plan?	Yes	71.74
		No	28.26
6)	Do you have adequate vaccine storage capacity?	Enough	43.48
		Limited	47.83
		Absent	8.7
7)	Are there sufficient basic facilities in place for the vaccination campaign for PPR control and eradication?		
	A. Vehicles	Enough	32.61
		Very Limited	76.39
	B. Cold chain and ice pack	Enough	54.35
		Very Limited	45.65
	C. Syringes and needles	Enough	80.43
		Very Limited	19.57
8)	Do you have suitable veterinary infrastructure?		
	A. Sample collection	Yes	52.17
		No	47.83
	B. Transport to laboratories	Yes	50.0
		No	50.0
	C. Implementation of diagnostic techniques	Yes	52.17
		No	47.83
	D. Data interpretation	Yes	60.87
		No	39.13
9)	Are there enough human resources for epidemiological surveillance and vaccination?		
	A. In numbers	Enough	41.3
		Moderate	47.83
		Limited	10.87
	B. In skills	Enough	41.3
		Moderate	50.0
		Weak	8.7
10)	Do farmers’ organizations exist?	Yes	65.22
		No	34.78
11)	How would you rate support from farmers for PPR eradication?	Enough	56.52
		Weak	43.48
		None	0.0
12)	Have you faced security problems that made field operations difficult or impossible?	Yes	65.22
		No	34.78
13)	Are PPR eradication efforts combined with control measures for other small ruminant diseases (provision of therapeutic services for the control of ecto and endo-parasites)?	Yes	65.22
		No	34.78

### Animal health professionals’ insights on gaps in monitoring and verification

AHPs reported that PPR vaccination campaigns face different challenges that limit effective disease control. A major constraint is the absence of markers to identify previously vaccinated animals, complicating follow-up and booster campaigns. They also highlighted information gaps due to the lack of post-vaccination serological monitoring, making it difficult to confirm vaccine effectiveness, cold chain maintenance, or development of protective immunity. Structural and behavioral factors further constrain vaccination. Unrestricted herd movement and rapid turnover of young stock hinder delineation of risk areas, leaving a persistent pool of susceptible animals. AHPs also noted technical and strategic limitations of the current RBVS: focusing on designated “high-risk” areas may miss susceptible animals in highly mobile populations, thereby reducing overall coverage and herd immunity.

## Discussion

FAO and WOAH launched the PPR GEP, which follows a four-stage, stepwise Global Control and Eradication Strategy (GCES) aligned with decreasing disease risk and increasing levels of control and verification^28^. Although global PPR eradication is technically feasible, poorly monitored vaccination can allow continued virus circulation and compromise control efforts^12,39,40^. Findings from the present study, complemented by additional data from the same project^41^, indicate that North Shewa Zone has made measurable progress along the GCES pathway, while key gaps in immunity and vaccination coverage persist, limiting advancement toward final verification.

Within the FAO–WOAH GCES, Stage 1 emphasizes comprehensive epidemiological assessment and risk characterization through systematic surveillance, outbreak investigation, and risk profiling^8^. Evidence from the present study demonstrates that North Shewa Zone has made substantial progress in fulfilling these requirements. Epidemiological assessments were implemented through routine outbreak reporting, laboratory confirmation using rapid antigen detection tests, and spatial analysis of outbreak-prone districts, consistent with previous studies^24,33^. These approaches enabled identification of high-risk districts with recurrent outbreaks and high animal movement, providing a solid evidence base for targeted control planning. Complementary serological investigations from the same project^41^, revealed moderate to high sero-positivity (22–100%) in several PAs that had not received vaccination, with district-level sero-positivity ranging from 48.2% to 92.6%. These results indicate ongoing silent virus circulation or subclinical infection rather than true disease absence, highlighting a limitation of outbreak-driven risk classification alone. Accordingly, while outbreak-based surveillance is well established in the study area, integration of serological data remains essential to fully meet Stage 1 objectives and refine risk profiling^8,9^.

Stage 2 of the GCES emphasizes implementation of control measures, particularly vaccination, supported by targeted surveillance^8^. Ethiopia adopted a national RBVS in September 2018, prioritizing high-risk areas identified through epidemiological evidence^20,24,33,42^. Findings from the present study demonstrate that this strategy has been operationalized in North Shewa Zone, where 62 PPR vaccination campaigns were conducted between September 2018 and August 2024. Districts such as Kewet accounted for a higher proportion of campaigns, consistent with their classification as high-risk areas based on outbreak history^24^. Overall, 2,917,330 small ruminants were vaccinated during the study period, including 1,284,339 sheep and 1,632,991 goats. Compared with other regions, such as Borena Zone (10,000 doses between 2018 and 2022)^20^ and South West Ethiopia Regional State (883,772 doses)^42^, North Shewa achieved relatively higher vaccination output. This substantial vaccination output reflects strong implementation of RBVS within prioritized districts, aligning with FAO–WOAH Stage 2 objectives^8,9^. Nevertheless, six districts did not implement any vaccination campaigns during the study period. While the absence of reported outbreaks partly explains this exclusion, serological evidence from the same project^41^ indicates that some unvaccinated areas maintain high immunity gaps. Similar challenges related to geographic inaccessibility and logistical constraints have been documented elsewhere^43,44^. FAO–WOAH guidance emphasizes that Stage 2 control requires proactive vaccination informed by surveillance, rather than reactive responses alone, to prevent undetected virus circulation and ensure equitable coverage^8,9^.

Stage 3 of the GCES focuses on reducing virus circulation to very low levels through sustained vaccination, achievement of herd immunity, and strengthened monitoring^8^. FAO–WOAH recommends achieving at least 80% population immunity to interrupt transmission^8^. Although the number of vaccinated animals in North Shewa reflects progress toward this stage, previously reported antibody levels (65.4%) from the same project^41^ remain below the recommended threshold. Evidence from Morocco indicates that herd immunity above 70% can substantially reduce virus transmission^45^, suggesting that improved coverage in North Shewa could yield meaningful epidemiological benefits. However, gaps in vaccination coverage and uneven campaign implementation continue to limit full interruption of transmission. Comparable findings from other localities of Ethiopia^20^, Somalia^46^, and Uganda^47^ show that sporadic outbreaks can occur even in areas with high vaccination output when coverage is uneven or campaigns are delayed. Similarly, exclusion of some districts from vaccination may increase vulnerability to resurgence, as observed in Tanzania in 2019, where immunity gaps led to a sharp increase in outbreaks^48^. Taken together, findings from the present study and the same project^41^ indicate that North Shewa Zone is progressing toward GCES Stage 3 but has not yet achieved sustained elimination. Continued implementation of RBVS, strengthened integration of serological and outbreak surveillance, and systematic immunity verification are therefore essential to advance Ethiopia toward PPR eradication and final verification stages^8,9^.

Stage 4 of the GCES requires countries to demonstrate absence of virus circulation through robust surveillance, PVS, and eventual cessation of vaccination to allow accurate verification for official PPR-free status^8,9^. Findings from this study indicate that North Shewa has not yet met Stage 4 requirements. AHPs identified the absence of PVS as a major constraint, limiting verification of antibody development, cold chain integrity, and true vaccination coverage. This aligns with previous studies emphasizing that sero-monitoring is essential for assessing immunity, identifying gaps, and guiding effective eradication strategies^49,50^. In addition, the lack of reliable animal identification prevents accurate recognition of vaccinated animals, complicating follow-up campaigns and interpretation of serological data^33^. Although ear tagging and ear marking are effective, their reliability depends on consistent application and long-term retention. As practical alternatives, temporary marking methods such as paint or dye application were recommended due to their low cost, ease of application, and visibility during mass campaigns^51^. The continued reliance on reactive vaccination further delays readiness for Stage 4, as FAO–WOAH guidance stresses that vaccination must only be halted once robust surveillance systems confirm absence of virus circulation^8,9^.

Understanding both the enabling factors and constraints within the existing system is critical for shaping future strategies that are practical, evidence based, and sustainable. Several key enabling factors support Ethiopia’s progression along the FAO–WOAH GCES pathway. A substantial proportion of respondents (82.6%) acknowledged the existence of a national PPR eradication policy, reflecting strong political commitment and institutional alignment with global eradication objectives. Such a supportive policy environment constitutes a fundamental prerequisite for advancing from control to eradication stages within the stepwise framework^52,53^. Confidence in vaccine production capacity at the National Veterinary Institute further strengthens the sustainability and continuity of national vaccination efforts.

Another encouraging finding is the availability of vaccination tools, with 80.43% of respondents reporting adequate supplies of syringes and needles, reducing logistical challenges. Moreover, 65.22% indicated that PPR eradication is integrated with other small ruminant disease programs, such as parasite control. Joint vaccination campaigns help lower costs, improve logistics, and increase coverage, while combining disease control efforts enhances overall efficiency and resource use^39,54^. Success further relies on local engagement, with health workers and veterinarians leading vaccination, surveillance, and education efforts^55^.

Community engagement is critical for PPR control and eradication. In this study, 65.22% of respondents reported that farmers’ organizations provide important platforms for mobilizing local participation, enhancing awareness, trust, and ownership of vaccination campaigns, supporting previous findings on the central role of community involvement^39^. Expanding education on vaccination, disease prevention, and early identification of PPR symptoms can further improve uptake and compliance^56^. Targeted programs for livestock owners strengthen grassroots cooperation and support vaccine delivery^28,57^. Moreover, 60.87% of respondents indicated adequate capacity for data analysis, enabling evidence-based decision-making, targeted interventions, and early outbreak detection, consistent with the FAO–WOAH Stepwise Monitoring and Evaluation framework^58^. These findings indicate that Ethiopia has established key elements necessary for progression along the FAO–WOAH PPR stepwise pathway, aligning with earlier studies emphasizing community engagement and technical capacity as critical for sustainable eradication^28,43^.

The survey revealed mixed findings regarding veterinary infrastructure, highlighting both strengths and critical gaps. Deficiencies in key areas were reported by approximately half of respondents, which can hinder effective disease monitoring and control. These findings align with previous studies showing that achieving herd immunity against PPRV is challenged by logistical constraints, limited veterinary services, and resistance to vaccination programs^28,59,60^. Major concerns identified include inadequate transportation and cold chain facilities, raising the risk of vaccine spoilage and compromising vaccination success^50,61^. Reliable vehicles and cold storage are essential, particularly in remote areas where small ruminants are predominantly kept. To address these challenges, mobile vaccination units and regional veterinary offices could improve outbreak response and coverage. Strengthening disease surveillance systems, including active case finding and rapid diagnostic testing, is also critical for early detection and rapid response, especially in regions with low vaccination coverage^8,58^.

Despite these enabling factors, significant challenges continue to undermine the effectiveness of eradication efforts, foremost among them being inadequate funding. While 82.6% of respondents acknowledged the existence of a national policy, only 10.87% considered the current budget sufficient, and 84.78% reported funding as limited. This reflects a broader issue identified in previous studies, where insufficient financing remains a major constraint to effective disease control^28,50,53^. This disparity underscores the gap between policy and financial commitment, a common issue in public health initiatives^50^. The economic burden of PPR, estimated at billions of dollars annually, calls for a reevaluation of funding priorities to ensure adequate resources are allocated for eradication efforts^39^.

Security challenges emerged as a major hindrance, with respondents reporting that safety concerns disrupted field operations (65.2%). This aligns with earlier studies showing the negative impact of conflict and instability on veterinary services and disease control^39^. In such regions, vaccination campaigns are often interrupted, exacerbating outbreaks and increasing economic losses, a challenge not unique to Ethiopia but shared globally in PPR eradication efforts^20,28,52,53^. Another constraint is the lack of transparency and communication from national authorities, reported by respondents (52.3%). Poor communication undermines trust and collaboration among stakeholders, highlighting the importance of clear, transparent messaging to promote community participation and ensure the success of disease control initiatives^28,39^.

Insights from AHPs revealed critical gaps in monitoring and verification that continue to hinder compliance with stepwise eradication requirements. In particular, respondents emphasized that vaccination efforts are frequently reactive, being implemented only after outbreaks occur, thereby leaving a persistent “window” of susceptible and unprotected livestock. This concern is consistent with evidence indicating that rapid population turnover, especially among young animals, significantly increases vulnerability to PPR outbreaks. Moreover, small ruminants are predominantly raised under extensive production systems by marginalized communities with limited access to veterinary services, and their short production cycles further complicate the implementation of routine health interventions^39^. Collectively, these findings highlight the need for proactive, regularly scheduled vaccination programs supported by integrated surveillance, systematic animal identification, and strengthened monitoring systems to sustain herd immunity and advance toward PPR eradication, in alignment with FAO–WOAH recommendations.

### Limitation of the study

A limitation of this study is that the potential role of wildlife and wildlife–livestock interfaces in the epidemiology of PPR was not investigated. Addressing this aspect could provide a more comprehensive understanding of disease dynamics in the study area.

## Conclusion and recommendations

This study demonstrates that the eradication of PPR in North Shewa Zone is biologically feasible and operationally achievable, with clear progress along GCES Stages 1–3. Since 2018, the national RBVS system has strengthened outbreak confirmation, targeted vaccination, and coordinated field operations. Capacity-building initiatives at the zonal, district, and peasant association levels have enhanced preparedness and technical competence. High vaccination outputs, together with supportive policy and institutional frameworks, indicate substantial advancement along the FAO–WOAH stepwise pathway. However, persistent challenges, including financial constraints, security-related access limitations, uneven vaccination coverage, the absence of systematic animal identification, and logistical gaps, continue to hinder progression to Stage 4 and verification of PPR-free status. Addressing these constraints is essential to consolidate existing gains and achieve elimination. To advance Ethiopia toward Stage 4 of the FAO–WOAH GCES and ensure sustainable PPR eradication, the following recommendations are proposed:

- Strengthen surveillance and PVS to improve early outbreak detection, assess immunity status, and guide more effective RBVS.
- Improve operational capacity and coordination by ensuring stable resource allocation, reinforcing supply chains, and promoting stronger collaboration across regional, district, and community levels.
- Implement context-appropriate field strategies that address security and logistical challenges to ensure continuous vaccination access in high-risk, underserved, and hard-to-reach areas.
- Enhance community engagement to encourage timely reporting, increase participation in vaccination campaigns, and raise awareness of PPR prevention and control.
- Expanding vaccination beyond outbreak-defined areas and targeting mobile or underserved populations.
- Implementing practical animal identification systems to support monitoring and follow-up campaigns.
- Conduct further investigations into the potential role of wildlife and wildlife–livestock interfaces in PPR epidemiology to achieve a more comprehensive understanding of disease dynamics in the study area.

## Supplementary Information

Below is the link to the electronic supplementary material.

Supplementary Material 1

Supplementary Material 2

## Data Availability

The data that support the findings of this study are available from the corresponding author upon reasonable request.
