# Supplementary material for: Vaccination trends and operational challenges in Peste des Petits Ruminants eradication in Ethiopia
Source: Sci Rep. 2026 Feb 27;16:11259. doi: 10.1038/s41598-026-41404-7 (PMC13049157; doi:10.1038/s41598-026-41404-7)
Supplement: Supplementary file 1 — Supplementary Material 1 [file 41598_2026_41404_MOESM1_ESM.pdf]

Supplementary Information (SI)

SI Annex 1a: Assessing Adequacy of the Risk-Based Vaccination Campaign (RBVC)

This questionnaire assesses the adequacy and implementation of the Risk-Based Vaccination Campaign (RBVC) for Peste des Petits Ruminants (PPR) control and eradication in North Shewa. It collects information on vaccination strategies, vaccine use, coverage, immune response monitoring, sample submission, and diagnostic practices to evaluate vaccination effectiveness and identify implementation gaps. Participation is voluntary, and respondents are requested to provide information based on their professional experience. If you agree to participate, please sign in the space provided below.

Name of the respondent_____________________ signature__________ Phone no. ___________

1. Did the global PPR eradication program start in the North Shewa zone? A. Yes B. No
2. If yes (Q1), when was the PPR-RBVC started? ___________
3. What type of vaccination strategy was implemented? __________________________
4. What types of vaccines were used? A. Thermo-stable B Thermo-labile
5. When were you vaccinated? (Months) ____________________________
6. At what interval did you vaccinate the same flock? ___________________________
7. How many districts are covered per year? ______________________
8. How many sheep and goats are vaccinated per year? ________________________
9. Did you check the immune response of the vaccine? A. Yes B. No
10. If yes (Q9), how many animals were sero-positive? ______________________
11. Did you submit samples for lab confirmation? A. Yes B. No
12. If yes (Q11); what types of samples were submitted A. Serum B. Swaps (nasal and lacrimal) C. postmortem (kidney, lung, spleen)
13. 15. If yes (Q12); How many samples were submitted, and when and where were they from? ____
14. What types of test /s has implementing? _______________

SI Annex 1b. Key Informant Interview Guide for RBVS Implementation

This key informant interview guide collects qualitative information on the implementation of the Risk-Based Vaccination Strategy (RBVS) for Peste des Petits Ruminants (PPR) control and eradication in North Shewa. It documents operational procedures, training practices, outbreak detection and response, prioritization of vaccination areas, and challenges encountered during field implementation. The information gathered supports evaluation of RBVS performance and helps identify operational gaps to strengthen vaccination strategies. Participation is voluntary, and respondents are requested to provide information based on their professional experience. If you agree to participate, please sign in the space provided below.

Name of the respondent____________________ signature__________ Phone no. ___________

1. Since when has RBVS been implemented in North Shewa? _________________
2. Can you describe the procedures followed before vaccination campaigns? ____________
3. What types of training are provided to animal health professionals prior to vaccination? ___
4. Which topics are commonly covered during these trainings? ________________________
5. How are suspected PPR outbreaks detected and reported? ___________________________
6. What methods are used to confirm outbreaks? ___________________________
7. What actions are taken once PPR is confirmed? ___________________________
8. Which vaccine is used for outbreak response? _____________________________
9. How are high-risk areas identified and prioritized? ________________________
10. How does vaccination differ between secure and insecure peasant associations? _________
11. What are the major challenges affecting RBVS implementation? ___________________
12. What improvements would you recommend? ___________________________________

SI Annex 2: Assessing Major Constraints on the Progress of PPR Eradication

This questionnaire aims to assess key constraints affecting the progress of the global PPR eradication program through RBVC at the zonal, district, and peasant association levels. It is designed to identify factors influencing the RBV implementation, examine drivers impacting vaccine coverage, and provide recommendations for corrective actions. If you agree to respond to the following questions, please sign it in the space provided below.

Name of the respondent____________________ signature__________ Phone no. ___________

1. 1. Is there a national policy for PPR eradication? A. Yes B. No
2. Do national authorities provide transparent information regarding the PPR situation?
   a. A. Yes B. No
3. Do you think that PPR eradication measures are organized and implemented in a coherent way? A. Yes B. No
4. Do you have sufficient national budget allocation for PPR eradication (free vaccine delivery for animal's owners (cost of vaccine), peridium, others logistic facilities)?
   a. A. Enough B. Limited C. Non-existent
5. Are there national laboratories capable of producing sufficient vaccines to meet the vaccination targets of the National Strategic Plan? A. Yes B. No
6. Do you have adequate vaccine storage capacity? A. Enough B. Limited C. Absent
7. Are there sufficient basic facilities in place for the vaccination chain for PPR control and eradication? I. Vehicles: A. Yes B. Very Limited
   a. II. Cold chain: A. Yes B. Very Limited
   b. III. Other vaccination equipment (syringes, needles, ice packs): A. Yes B. Very Limited
8. Do you have suitable veterinary infrastructure?
   a. I. Sample collection: A. Yes B. No
   b. II. Transport to laboratories: A. Yes B. No
   c. III. Implementation of diagnostic techniques: A. Yes B. No

d. IV. Data interpretation: A. Yes B. No

9. Are there enough human resources for epidemiological surveillance and vaccination?
   a. I. In numbers: A. Enough B. Moderate C. Limited
   b. II. In skills: A. Enough B. Moderate C. Weak
10. Do farmers' organizations exist? A. Yes B. No
11. How would you rate support from farmers for PPR eradication?
    a. A. Enough B. Weak C. None
12. Have you faced security problems that made field operations difficult or impossible?
    a. A. Yes B. No
13. Are PPR eradication efforts combined with control measures for other small ruminant diseases (provision of therapeutic services for the control of ecto and endo-parasites)? A. Yes B. No
14. Based on your experience, what are the main challenges affecting the effectiveness of PPR vaccination campaigns in this region? Please describe key economic, logistical, operational, behavioral, or other factors, and explain how they influence vaccine coverage, herd immunity, and overall disease control. ____________________________________
