# Supplementary material for: Vaccination trends and operational challenges in Peste des Petits Ruminants eradication in Ethiopia
Source: Sci Rep. 2026 Feb 27;16:11259. doi: 10.1038/s41598-026-41404-7 (PMC13049157; doi:10.1038/s41598-026-41404-7)
Supplement: Supplementary file 2 — Supplementary Material 2 [file 41598_2026_41404_MOESM2_ESM.docx]

**Supplementary Information (SI)**

**SI Annex 1a** is designed to evaluate the effectiveness of the PPR RBVS, covering campaign initiation, vaccination strategy, vaccine types, schedules, coverage, and immune response testing.

**SI Annex 1b** is designed as a Key Informant Interview guide to collect information on RBVS implementation in North Shewa, including pre-campaign procedures, training, outbreak detection and confirmation, vaccination approaches, prioritization of high-risk areas, operational challenges, and recommendations.

**SI Annex 2**: This annex aims to identify and assess constraints that may hinder the progress of the global PPR eradication program. It includes a comprehensive questionnaire that explores challenges at the zonal, district, and peasant association levels. Topics include national policy and legal frameworks, government support, vaccine availability and storage, veterinary infrastructure, and human resource capacity for epidemiological surveillance and vaccination. It also assesses farmers' support, security issues, and coordination with other small ruminant disease control efforts.
